# Implementation of tobacco cessation brief intervention in complementary and alternative medicine practice: qualitative evaluation

**DOI:** 10.1186/s12906-017-1836-7

**Published:** 2017-06-23

**Authors:** Emery R. Eaves, Amy Howerter, Mark Nichter, Lysbeth Floden, Judith S. Gordon, Cheryl Ritenbaugh, Myra L. Muramoto

**Affiliations:** 1 Department of Anthropology, Northern Arizona University, 5 E. McConnell Drive, PO Box: 15200, Flagstaff, AZ 86011-5200 USA; 2 Department of Family and Community Medicine, College of Medicine, University of Arizona, 1450 N Cherry Ave, Tucson, AZ 85719 USA; 3 School of Anthropology, College of Social and Behavioral Sciences, Department of Family and Community Medicine, College of Medicine, University of Arizona, P.O. Box 210030, Tucson, AZ 85721-0030 USA; 4 Department of Pharmacy Practice & Science, College of Pharmacy, University of Arizona, 1295 N. Martin, PO Box 210202, Tucson, AZ 85721 USA

**Keywords:** Tobacco cessation, Behavioral intervention, Complementary and alternative medicine, CAM

## Abstract

**Background:**

This article presents findings from qualitative interviews conducted as part of a research study that trained Acupuncture, Massage, and Chiropractic practitioners’ in Arizona, US, to implement evidence-based tobacco cessation brief interventions (BI) in their routine practice. The qualitative phase of the overall study aimed to assess: the impact of tailored training in evidence-based tobacco cessation BI on complementary and alternative medicine (CAM) practitioners’ knowledge and willingness to implement BIs in their routine practice; and their patients’ responses to cessation intervention in CAM context.

**Methods:**

To evaluate the implementation of skills learned from a tailored training program, we conducted semi-structured qualitative interviews with 54 CAM practitioners in Southern Arizona and 38 of their patients. Interview questions focused on reactions to the implementation of tobacco cessation BIs in CAM practice.

**Results:**

After participating in a tailored BI training, CAM practitioners reported increased confidence, knowledge, and motivation to address tobacco in their routine practice. Patients were open to being approached by CAM practitioners about tobacco use and viewed BIs as an expected part of wellness care.

**Conclusions:**

Tailored training motivated CAM practitioners in this study to implement evidence-based tobacco cessation BIs in their routine practice. Results suggest that CAM practitioners can be a valuable point of contact and should be included in tobacco cessation efforts.

**Electronic supplementary material:**

The online version of this article (doi:10.1186/s12906-017-1836-7) contains supplementary material, which is available to authorized users.

## Background

Tobacco use is the leading cause of preventable death in the United States [1–4]. Research has shown that receiving cessation advice from any health care professional increases quit rates [5–7] and that advice and support from more than one type of health professional can substantially increase readiness to quit [8–10]. The present study aimed to assess the feasibility and impact of training complementary and alternative medicine (CAM) practitioners’ (Chiropractors, Licensed/Certified Massage Therapists, and Acupuncturists) to become part of such a network of health professionals providing cessation intervention. CAM practitioners were provided with a tailored training in implementing a tobacco cessation brief intervention (BI) in their routine practice that included guiding patients towards evidence-based cessation tools like quitlines or nicotine-replacement options. This article reports the results of qualitative evaluation of the implementation of tobacco cessation BI in CAM practice following tailored training. Results included the perspectives of practitioners that participated in the study and their patients.^1^

Increases in tobacco screening initiatives have improved practitioner screening for tobacco use [11, 12] and there has been some movement in overall quit rates in the US [13], however, a substantial number of adult smokers continue to struggle to remain tobacco free. Health care practitioners are unlikely to assist with tobacco cessation if they are not confident in their ability and knowledge to assist [14–16] and many report time constraints as the biggest barrier to effectively assisting a patient with tobacco cessation [11]. Although tobacco cessation BI training for both health care practitioners and laypersons can effectively extend the reach of tobacco intervention [17, 18], training initiatives are almost exclusively focused on conventional medical professionals in clinical settings [19, 20]. Our training was designed in accordance with the 2008 Clinical Practice Guideline for Tobacco Use [21] to increase the number of contacts with health professionals screening for tobacco use, assessing for readiness to quit, and utilizing motivational interviewing techniques to encourage patients who are resistant to cessation attempts. Compared to biomedical practitioners, interactions with CAM practitioners are typically longer, more frequent, and more focused on wellness care [22]. Further, a considerable proportion of US adults (33.2% in 2012), [23] including approximately 1.5 million smokers, seek care from CAM practitioners annually [24, 25]. The potential reach of CAM practitioners in promoting tobacco cessation has unfortunately been largely overlooked by cessation initiatives [15, 26]. Following the successful implementation of the CAMR training intervention, this paper presents the results of qualitative interviews assessing the implementation of tobacco BIs in CAM practice from the perspectives of both practitioners and patients.

## Methods

Qualitative results presented in this article were collected as part of a larger study, CAM Reach (CAMR) (NCI RO1 CA137375) [26, 27], that trained Acupuncture, Chiropractic, and Massage Therapy practitioners to implement tobacco cessation brief intervention (BI) in their practice. The training was specifically tailored and adapted for use in CAM practice settings.

Prior to CAMR intervention development, investigators conducted semi-structured, open-ended qualitative key informant interviews with local CAM practitioners chosen for expertise in their discipline and connectedness with professional peers (see Muramoto et al. [26] for a description of these interviews as part of the intervention development process, including themes covered, participant quotations, and in-depth interviewing methods).

Key informants in those interviews raised three primary concerns [26] that would be addressed in the CAMR study:

- First, that regularly raising the issue of tobacco cessation might compromise their relationship with patients, particularly those whose key complaints may not be seen by the patient as tobacco-related.
- Second, given that most patients pay for CAM services out-of-pocket, practitioners were concerned that promoting cessation might cause patients to leave their practice.
- Third, practitioners were concerned that introducing cessation materials, such as pamphlets or posters, to new patients might be perceived as a “sales pitch” aimed at promoting additional, fee-based products or services [26].

Four months after CAMR training, a second phase of qualitative interviews assessed practitioners’ experiences after participating in the training. The purpose of these qualitative interviews was to determine whether tailoring BI training to address key informants’ concerns was effective in making the BI a routine and accepted part of CAM practice. This article is focused on analysis of data from the second phase of qualitative inquiry, conducted with the CAMR study practitioners and their patients after participation in tailored training.

The CAMR training and practice system intervention (CAMR Study intervention, Table 1) tailored BI training to the everyday reality of CAM practice settings with specific emphasis on addressing potential concerns. The goal of the training was to increase practitioners’ sense of self-efficacy and comfort in initiating BIs by teaching practitioners to deliver tobacco cessation advice to patients in non-intrusive ways that could be easily incorporated in their practice.

**Table 1:** Summary of CAMR study

Overall objective	▪ Train chiropractors, acupuncturists and massage therapists to perform tobacco BIs with their patients
Aims	▪ Evaluate the effectiveness of the CAMR training by studying practitioner BI behavior and patient tobacco cessation activity. ▪ Conduct a qualitative study with a sub-sample of enrolled practitioners and patients to examine factors associated with implementing tobacco cessation BIs
Design and methods	▪ Single group, Pre/post post design; assessments measured knowledge, attitudes, beliefs and confidence about tobacco BIs (assessed at baseline, and 3-, 6-, 9- and 12-months post-training). ▪ Training was a one-day, in-person workshop and included a one-hour in-situ “practice patient” learning activity 1–2 weeks later. ▪ Practitioners were provided tobacco cessation materials (pamphlets and posters) to display in their practices and distribute to their patients. ▪ After the CAMR training, research staff visited practices every 2–4 weeks for 3 months, to encourage practitioners to incorporate study patient materials and implement tobacco cessation activities into routine practice. ▪ *N* = 99 practitioners (30 chiropractors, 27 acupuncturists and 42 massage therapists) enrolled in the study. ▪ *N* = 595 clients of enrolled practitioners participated in the study. ▪ Of these participants, 54 practitioners and 38 patients were selected for qualitative interviews spread across all three cohorts.

### Qualitative methods

The qualitative component of the CAMR study was designed and conducted by an interdisciplinary team of public health and medical researchers and medical anthropologists. It consisted of telephone interviews with practitioners and patients ranging from 10 to 55 min in length. Semi-structured interview guides allowed interviewers to explore the concerns raised during key informant interviews [26] and to cover other aspects of their training and experience, but included some set questions that all participants were asked to respond to (See Additional file 1).

#### Practitioner interviews

Practitioners from all three cohorts (chiropractic, acupuncture, massage therapy) who participated in CAMR training were eligible to participate in qualitative interviews based on self-reported tobacco cessation BI- related activity in the past 3 months. BI-related activity indicators were: (1) reports of BIs with patients who use tobacco; (2) distribution of tobacco cessation related materials; (3) referral of patients to Public Health Service guideline-based tobacco cessation treatment; and/or (4) conversations with non-tobacco users exposed to second-hand smoke. Interview questions asked about interactions with both established and new patients, the contexts in which conversations about tobacco were introduced, patients’ reactions to the discussion, and the use of tobacco related educational materials.

#### Patient interviews

Patients were eligible to participate in qualitative interviews if they had visited their practitioner within the previous three months and reported conversations with their practitioner about tobacco use or second-hand smoke. Patient interviews focused on how appropriate or intrusive it was for CAM practitioners to ask about tobacco use; changes in patients’ thinking about tobacco use following BIs; and how CAMR patient education materials were used or passed on.

### Qualitative participants

Of 99 practitioners and 595 patients enrolled in the study, 54 practitioners and 38 patients participated in qualitative interviews (see Table 2 for number eligible based on meeting criteria of having conducted a BI in the past 3 months, and response rates). Optimally, practitioners were interviewed multiple times, at 3 months, 7 months, and 1 year post-training to assess implementation over time. Patients were interviewed once to assess perception of the brief intervention delivered by a CAM practitioner, and those who reported intention to quit or active quit attempts discussed with practitioners were interviewed again three months later. One hundred and one practitioner interviews and 50 patient interviews were included in this analysis.

**Table 2:** Numbers of practitioners and patients eligible to participate in qualitative interviews and response rates among those eligible^a^

		Months 3–4	Months 7–9	Month 12	Total
Practitioners	# eligible	*n* = 45	*n* = 45	*n* = 43	*n* = 133
	# completed	*n* = 38	*n* = 32	*n* = 31	*n* = 101
	Response rate	84.4%	71.1%	72.1%	75.9%
Clients	# eligible	*n* = 13	*n* = 37	*n* = 21	*n* = 71
	# completed	*n* = 10	*n* = 26	*n* = 14	*n* = 50
	Response rate	76.9%	70.3%	66.7%	70.4%

^a^Practitioners were eligible to participate in qualitative interviews if they reported having discussed tobacco use or second-hand smoke with patients during their interactions in the past 3 months. Patients were eligible if they reported having seen tobacco-related materials or having discussed tobacco use or second-hand smoke with practitioners in the past 3 months

### Qualitative analysis

Semi-structured qualitative interviews were audio-recorded and transcribed verbatim. The research team analyzed qualitative data using constant comparative analysis [28, 29] and phenomenological analysis methodologies [30, 31]. The coding scheme used to code data was based on initial thematic analysis and additional codes were added when new themes became apparent. In-depth thematic coding was conducted using ATLASti qualitative data analysis software [32]. Thematic analysis of data included a “mechanical” stage (organizing and dividing the data into a useful scheme/codebook), and an “interpretive” stage (identifying criteria for organizing thematic codes) [33]. Phenomenological analysis is a method of contextualizing participants’ experiences and gaining in-depth understanding of how their interpretation of the meaning in their experiences impacts behavior [30, 31].

Six coders were involved in coding the transcripts. Reliability among coders was established through a process of regular meetings of all coders during which they discussed differences in their coding of a single transcript until there was consensus among coders as to the definition and scope of the themes. This reliability procedure was intended to critically reflect on disagreements and improve analytical consistency, not to measure percentage of agreement [34, 35]. Coded qualitative data were analyzed by a researcher not involved in the study design or coding process (EE) to reduce potential bias in results that can be produced when initial expectations of interviewers drive themes [35]. Using thematically grounded qualitative analysis, the research team was attentive to both emergent themes and topics of interest such as how practitioners and patients engaged with the topic of tobacco use and second hand smoke exposure.

## Results

The CAMR training and practice system intervention (CAMR Study intervention, Table 1) tailored BI training to the everyday reality of CAM practice settings with specific emphasis on addressing potential concerns. The goal of the training was to increase practitioners’ sense of self-efficacy and comfort in initiating BIs by teaching practitioners to deliver tobacco cessation advice to patients in non-intrusive ways that could be easily incorporated in their practice.

We first highlight findings from qualitative interviews specifically addressing the three concerns described by the initial key informants. Strategies found useful in implementing tobacco BIs in routine CAM practice are then briefly considered.

### Concern 1: Potential harm to the patient-practitioner relationship

#### Practitioners’ perspective

Prior to the training, even if questions about tobacco were included on patient intake forms, practitioners rarely followed up on reports of tobacco use. After the training, however, practitioners reported greater understanding of the connections between tobacco use and common patient complaints (e.g. pain, impaired healing). Feeling more able to describe the relevance of their cessation advice increased practitioners’ confidence and motivation to discuss tobacco cessation. One practitioner, for example, explained that learning about how tobacco use impacts tissue healing motivated her to address tobacco with a patient.

I was more comfortable with learning how to put together the detriments of smoking, linking it with something that would be occurring. For instance, a massage client who has a stiff shoulder and upper back issues. He’s a smoker and through the training I learned that because smoking inhibits some of the circulation and it doesn’t allow muscles and tissue to heal as readily as a nonsmoker, I was able to bring that up. (Massage Therapist, 4 months post-training)

Connections between tobacco and pain were described as a helpful way to begin a BI (i.e. introduce the topic of tobacco) and as a key motivation to address tobacco use with patients.

I didn’t realize that tobacco was tied to pain levels. I didn't realize that people would not heal as quickly if they smoked. It’s good to be able to point that out to patients, that we’ve been working on this problem and some of the reason you still have pain could be because you’re still smoking. (Acupuncturist, 4 months post-training)

#### Patients’ perspective

CAMR BI training encouraged practitioners to assess patients’ readiness to quit and to support patients accordingly. If a patient had not thought about quitting or was contemplating a possible quit, for example, information was offered about both the general harm of smoking and possible links between smoking and current health problems. If and when a patient was ready to take steps toward planning or executing a quit attempt, practitioners offered practical advice, including referral to an Arizona statewide quit line (ASHline) where patients could obtain behavioral support and information about medications that might help with the process of quitting. Practitioners were taught to offer advice in a motivational, supportive manner and to refrain from proscriptive advice. Patients corroborated this in descriptions of how practitioners approached them about tobacco use. Notably, this approach did not compromise, but rather strengthened practitioner-patient relationships and was viewed as an act of caring. Patients who were interested in quitting tobacco reported that their practitioners were a nonjudgmental source of information and support for achieving their goals. One patient, for example, identified this approach to behavior change as empowering.

She made me feel empowered. She made me feel whole, that it was okay where I was right there, and that I could move forward to what I really truly wanted. It was just important that I really wanted it. It had to be a conscious choice of mine that I wanted it, not somebody else wanted it. … I just felt empowered—empowered and accepted, and not judged at all. (Acupuncture patient, 12 months post-training)

### Concern 2: Intrusiveness of BI in CAM practice

#### Practitioners’ perspective

Several practitioners in this study reported that prior to the CAMR training they were apprehensive about approaching patients about tobacco use. After participating in the training, however, they described being far more comfortable approaching patients using the skills they had learned. Practitioners explained that learning to approach tobacco-users as a source of information and referral gave them the confidence to approach patients. Also notable, as illustrated in the following quote, practitioners reported surprise that tobacco users were less defensive about talking about tobacco than they had anticipated.

It surprised me that it wasn’t as difficult as you think. The resistance isn’t there as much as you think it is. Because with the training, with the material, it’s not as much as like a parent to a child lecture. It’s more like, “Here, I want to help you,” and we’ve got tools. (Chiropractor, 7 months post-training)

As one practitioner explained, the training helped practitioners put aside personal biases and approach tobacco-addicted patients with empathy and support.

I’m definitely more willing to bring up tobacco outside of the initial intake form. I’m more willing to—more able to talk to people about it. Before, I had a hard time because I’m not a smoker, and I don’t like smoking. I don’t enjoy it in any sense, anywhere for anybody, and so I always felt like I maybe would come off as a little too biased and a little too headstrong, and so I was hesitant because I didn’t want to offend anybody, but now I feel a lot better about it. (Chiropractor, 12 months post-training)

#### Patients’ perspective

Contrary to Practitioners’ concern that patients might resist tobacco intervention, patients noted that wellness and lifestyle recommendations from their CAM practitioners were an expected part of seeking these therapies. This discrepancy between practitioners’ concerns and patients’ expectations regarding tobacco intervention has also been reported in dental and primary care settings [36, 37]. As one patient reported, not only was a frank discussion about tobacco use seen as an acceptable part of a CAM practice, it was valued as an important part of a wellness consultation

Yes, it’s appropriate… It definitely adds value to going to the appointment; I’m having the therapy because it’s more than just the massage. It’s also how that affects your entire life and what things you can do to achieve more benefits from the massage. (Massage client, 4 months post-training)

### Concern 3: The possibility of cessation support being perceived as a “sales-pitch”

#### Practitioners’ perspective

As part of the training, practitioners were offered continuing education credits and training completion certificates. Although some did not see value in a certificate that did not contribute to their licensing, most described the credential as increasing their legitimacy and patients’ receptivity to the information they offered.

It gives it a professional look rather than just me by myself saying, “Oh, you should think about quitting.” It gives me the nice documentation and presentation look of it: that I did go through training, I did learn about this, I did take continuing education on it, and it is through a well-known university, University of Arizona… A lot of them say, “Oh, the University of Arizona does this. Oh, that's so neat.” I guess it validates the information, and it gives it substance. (Licensed Massage Therapist (LMT), 7 months post-training)

Printed educational materials given to practitioners as part of the CAMR training figured prominently into practitioners’ descriptions of implementing the BI. Instead of seeming like a sales-pitch for additional services, the materials, in combination with techniques for non-confrontational communication provided as part of the training, were a way to provide information about the benefits of quitting tobacco without being pushy or discouraging patients from returning.

I’ve noticed that having the pamphlets available to the patient makes it a lot easier because it’s a way of conversing about the different aspects of quitting and having medications available, having ASH Line available. Then being able to just—if they’re not real open to it, just say, “Okay, well, here, take these. If you’d like to continue the conversation, we can. I’ll follow up later.” (Acupuncturist, 12 months post-training)

The CAMR training addressed a broad range of tobacco-related topics including availability of the state quit line (ASHline), how to refer patients for quit coaching, and basic information about cessation medications should they be asked direct questions by patients (e.g. most commonly used, which are available without prescription, referring patients to pharmacists or physicians for more information about cessation medications). This knowledge of quit-related resources further increased practitioners’ credibility as sources of cessation advice for patients who were interested in their assistance.

#### Patients’ perspective

When asked whether they felt the BI was an appropriate part of CAM therapeutic interaction, patients reported that rather than feeling pressured to accept—or pay for—additional forms of treatment, patients saw CAM practitioners as resources for wellness information and support. For example, a long time massage patient expressed initial concerns about her practitioner becoming involved in tobacco BI training. After seeing the materials offered in a supportive way, however, she revised her initial impression and described the information on quitting smoking as an additional form of wellness support.

[The practitioner] has a little area where people sit when they first come in. I thought, “Oh, God, is she gonna start advertising with us, and hitting us with stuff?” … At first it kind of put me off. Then I realized that it was yet another avenue of talking about a healthy body and keeping one’s body healthy. Actually, I really sort of admired her for doing that. (Massage Client, 4 months post-training)

### Strategies for implementing brief tobacco intervention in routine CAM practice

CAMR training was designed to complement the holistic approach common to all three types of CAM practice in the study. Practitioners were offered evidence-based techniques for effective communication and behavioral intervention. All three types of practitioners reported that as a result of the CAMR training, they were more likely to discuss tobacco use with patients. The training provided strategies for approaching patients in non-judgmental and supportive ways. Practitioners mentioned that the availability of pamphlets, posters, and certificates of training completion all enhanced their credibility as sources of tobacco cessation information. This identity contributed to their readiness to initiate discussions about tobacco use, pass on information, and make referrals to cessation services.

I’ve always asked if they smoked before [CAM] Reach, but now I have the education and the resources to follow up and do something about it rather than it just being another question on the intake form. I believe that it will remain the same, and I believe I’ll still be following up with clients and asking them because it seems to give my clients and I a closer relationship as far as their health is concerned. They share more when they realize I care about everything, not just coming in and giving a massage and leaving. (LMT, 12 months post-training)

As practitioners became aware of tobacco’s centrality to treating common ailments, they described increased motivation to engage in cessation efforts as a core means to achieve wellness goals.

I have to admit that I really did not pursue [tobacco] until I became a part of this [study]... I really did not pursue it. Now it’s just a regular part of my history taking… I love being a source of information for people that are interested in how we can live healthier lives, so that’s perfect for me. (Chiropractor, 3 months post-training)

CAM practitioners were trained in how to answer basic questions about nicotine replacement and other tobacco cessation medications, but reported being asked infrequently about them. They were, however, grateful to have this information included as part of the training as it boosted their confidence to talk to patients and enabled them to be seen as credible sources of information and referral. CAM practitioners who had been asked about cessation medications generally referred patients to primary care physicians and provided other types of support during the quitting process.

Practitioners noted that referring patients for cessation services did not constitute a statement about the limitations of their discipline, but rather that CAM practice is a site for making contact with and advising patients. If other support is required, CAM providers who are trained in tobacco cessation can advise, and in some cases arrange, for additional support, information, and resources.

Acupuncture is much better at treating other things than tobacco cessation. The way I would look at it is that at least if somebody were willing to come in for tobacco cessation, it means that they’re thinking of quitting. That, whether it’s acupuncture or anything else, is a good thing. (Acupuncturist, 4 months post-training)

## Discussion & Conclusion

Despite interest in wellness promotion and contact with large numbers of people seeking wellness care in the U.S., CAM practitioners are rarely included in tobacco cessation initiatives [24]. CAM practitioners are well-positioned to encourage tobacco cessation [38] and contrary to popular misconceptions, see about the same proportion of smokers as do other types of health professionals [24]. When compared with typical biomedical consultations, consultations with CAM providers are longer, more frequent, and more wellness focused [39]. These qualitative data collected as part of the CAMR study found that when offered the opportunity to be trained in evidence-based tobacco cessation BIs, CAM practitioners were interested in learning BI skills and implementing them in tobacco-cessation [26, 27]. This analysis of the implementation of tobacco cessation BI in routine CAM practice after participation in the CAMR study training suggests that it is feasible and acceptable to Chiropractic, Massage, and Acupuncture practitioners as well as to their patients who use tobacco.

After participation in tailored tobacco cessation intervention training as part of the CAMR study, practitioners reported confidence, knowledge, and motivation to address tobacco use. Based on their experience, practitioners described positive responses from patients regardless of their readiness to quit. In study interviews, patients reported openness to being approached by CAM practitioners about tobacco use and saw such inquiry as part of highly-valued, patient-centered care and a holistic approach to wellness. Cessation materials and certificates of training from a respected university increased CAM practitioners’ credibility as knowledgeable cessation resources. These qualitative findings suggest that with training tailored to their practice-related concerns, CAM practitioners can be valuable points of contact for reaching patients who desire help with tobacco cessation.

## Endnote

^1^Note that chiropractors and acupuncturists typically refer to persons receiving their treatments as “patients”, whereas massage therapists usually use “clients”. For simplicity, in this paper we use “patients.”

## Additional file

Additional file 1: CAM REACH Study (Muramoto NCI RO1 CA137375) Practitioner Qualitative Interview Guide. (DOCX 29 kb)

## Abbreviations

BI: Brief intervention
CAM: Complementary and alternative medicine
CAMR: CAM Reach Study
LMT: Licensed massage therapist
