# Supplementary material for: Implementation of tobacco cessation brief intervention in complementary and alternative medicine practice: qualitative evaluation
Source: BMC Complement Altern Med. 2017 Jun 23;17:331. doi: 10.1186/s12906-017-1836-7 (PMC5481908; doi:10.1186/s12906-017-1836-7)
Supplement: Additional file 1: — CAM REACH Study (Muramoto NCI RO1 CA137375) Practitioner Qualitative Interview Guide. (DOCX 29 kb) [file 12906_2017_1836_MOESM1_ESM.docx]

**CAM REACH Study (Muramoto** NCI RO1 CA137375)

**Practitioner Qualitative Interview Guide**

*Thank you for taking the time to share your opinions and experience with me. The following interview is intended to collect information from practitioners involved in Project Reach. The questions that follow will include items about yourself, your practice and how you interact with patients/clients. If you have any additional questions or comments, please feel free to share them with me. This interview will take approximately 30-45 minutes. We are also asking for your permission to audio tape this conversation. You can still participate in this interview if you decline to allow this conversation to be audio taped. Audio taping allows us to make a transcript, which is a more accurate record of our conversation. We then analyze the transcript to determine the results of the study. Any information that might identify you will be deleted from both the written and/or audio record. After the audio tape has been transcribed, we will destroy it. Can I have your permission to audiotape this conversation? <If participant declines, do not begin audio taping. If participant agrees, begin audio taping>.*

SECTION 1

1. How often do you discuss lifestyle issues with your patients (such as diet, exercise, tobacco, alcohol, sleep, etc.)?

Prompt: What is most common?

How do you go about starting these conversations?

How do your patients react when you talk with them about lifestyle issues?

1. During training, we taught you some strategies – identifying readiness, motivators and barriers -- have you used them for discussing lifestyle issues? How did it work?

Prompt: Which ones were you most comfortable with? Least comfortable with?

How did the patient/client react?

Is there anything about your interactions with patients when using these strategies that surprised you?

1. How do you currently document tobacco use?

Prompt: Has this changed recently or since the training?

Do you think patients expect to hear about tobacco from their practitioner?

1. After training have you tried to fit conversations about tobacco into your practice?
   1. If YES, continue.

Prompt: If you were already addressing tobacco use before the training, how has anything changed post-training?

At what point are you now introducing the tobacco discussion with your patients?

If applicable, compared to before?

How about e-cigs?

Does this differ by type of patient – your relationship with them, their illness, how much they say they smoke? Opening lines?

What would it take to keep you involved in talking about tobacco to clients?

Do you approach tobacco differently than other lifestyle issues (diet, nutrition, exercise)? Why or why not different?

Has this changed due to their participation in project?

Have you had conversations within social network and how did those go?

**If NO, skip to SECTION 4**

SECTION 2

1. Any positive and/or negative interactions? What advice/materials did you offer (referrals, services, medication handout, etc.)? How have you followed up/recovered?

Prompt: How did you go about starting the conversation? How easy or comfortable? How difficult or uncomfortable?

Have you engaged/encouraged patients to do a quit plan, why or why not?

Did you refer anyone to ASHLine…via quitfax or just used the handout? Why one or the other?

Would it help if we provided more copies of Quitfax?

Did you use medications handout, why or why not?

E-cig hand out and in what context?

In addition to what the training offered, are you doing anything to help clients with tobacco cessation?

What therapies are you using to alleviate symptoms of tobacco if any?

FOR MOBILE PRACTITIONERS ONLY: What materials do you travel with? How do you set up? Mobile versus non-mobile practice if they have both – what’s different in going to client home versus if they come to practice (relationship, what they talk about, etc)?

1. During training, we taught you some strategies – identifying readiness, motivators and barriers -- have you used them for tobacco? How did it work?

Prompt: Which ones were you most comfortable with? Least comfortable with?

How did the patient/client react?

Is there anything about your interactions with patients when using these strategies that surprised you?

1. How have the posters/pamphlets worked in making you more comfortable with brining up tobacco? Have patients brought it up themselves?

Prompt: How did you fit these changes into your practice to make them work?

How are you integrating the project handouts with what you have already been doing for tobacco cessation? E-cigs (if applicable)?

How have your patients/clients reacted to the new materials?

How have the posters/pamphlets helped your conversations with new patients?

How have the posters/pamphlets’ helped your conversations with established patients?

Do you plan to keep these materials out after the project ends?

1. Have more smokers entered your practice since you took part in the training? Did you seek them out or did they find you through referral from others?

Prompt: How have you advertised yourself as a resource for tobacco cessation since taking part in the training? Do you see yourself continuing to do so?

1. Were you concerned that brining up tobacco may be uncomfortable for patients and could harm the practice? Is this still a concern? Why (or why not) ?
2. Have you had a conversation with someone who was concerned about a friend or family member who smokes? If YES, How did that conversation get started?

Prompts if needed: How easy or difficult? If you talked about health effects of tobacco, what specifically did you address?

How did the patient/client react?

Did you refer clients to Helpers? How comfortable are you in referring clients to Helpers?

IF NO, how can SHS exposure questioning be prioritized higher in your practice? Would you add SHS exposure question to intake?

SECTION 3

Now I’d like to ask you a few questions about the Reach Training…

1. What parts of the training were most useful? Least?

Prompt: How has the training affected the way you talk to patients? (specifically, about behavior change)? How have you changed they way you offer advice about tobacco since the training?

What could the training have better prepared you for?

In regards to SHS conversations, what could we have done or provided in the training to better help you integrate it into your practice?

How can the Reach team better support you to integrate tobacco helping conversations into your practice?

1. What materials provided at the training were most useful? Least? Is there anything else you would like to have?

Prompt: What handouts did you distribute the most? The least?

Was it better to have a variety of handouts, allowing you to pick and choose the ones you liked? Does having these materials make talking about tobacco seem more like part of your role as a practitioner?

Do you plan to keep these materials out after the project ends?

1. What advice would you give other practitioners who have gone through the training? Have you shared the training with other practitioners?

Prompt: How does your support of tobacco cessation impact the way others look at your profession?

Do you feel your profession has a public health as well as a medical/healthcare role?

If yes --what do you see as the public health contribution of your profession?

How do you think smoking cessation could be an important part of your profession’s public health agenda?

Definition of Public Health: It is the practice of preventing disease and promoting good health within groups of people.

Now I would like to ask you some questions about yourself….

1. Do you currently smoke or use tobacco? Can you please describe your smoking/tobacco use to me? How has participating in this study influenced your tobacco use? Quit attempts?

SECTION 4

IF NOT having conversations or are using passive approach:

1. Why aren’t you having them? Concerns about offending patients?

Prompt: Do they approach tobacco differently that other lifestyle issues (diet, nutrition, exercise)? Why or why not different?

1. Is this something you think you will do in the future? Are you confident/comfortable that you could do this if/when you decided to start?
2. What do you think will get you to have these conversations?

Prompt: Once started what would it take to keep you involved in having these conversations?

1. If materials are around, to what extent are you waiting for patients to approach you?

Now I’d like to ask you a few questions about the Reach Training…

1. What parts of the training were most useful? Least?

Prompt: How has the training affected they way you talk to patients? (specifically, about behavior change)?

How have you changed they way you offer advice about tobacco since the training?

What could the training have better prepared you for?

In regards to SHS conversations, what could we have done or provided in the training to better help you integrate it into your practice?

How can the Reach team better support you to integrate tobacco helping conversations into your practice?

1. What materials provided at the training were most useful? Least? Is there anything else you would like to have?

Prompt: What handouts did you distribute the most? The least?

Was it better to have a variety of handouts, allowing you to pick and choose the ones you liked? Does having these materials make talking about tobacco seem more like part of your role as a practitioner?

Do you plan to keep these materials out after the project ends?

1. What advice would you give other practitioners who have gone through the training? Have you shared the training with other practitioners?

Prompt: How does your support of tobacco cessation impact the way others look at your profession?

Do you feel your profession has a public health as well as a medical role?

If yes --what do you see as the public health contribution of your profession?

How do you think smoking cessation could be an important part of your professions public health agenda?

Now I would like to ask you some questions about yourself….

1. Do you currently smoke or use tobacco? Can you please describe your smoking/tobacco use to me? How has participating in this study influenced your tobacco use? Quit attempts?

*This concludes my questions. Before we end, are there any final thoughts or comments you about your experiences with patients in your office that you would like to add?*

*Thank you very much for your time and input. You will receive your compensation from this interview in about 6-8 weeks. This is a ground-breaking study, and your participation is critical to the study’s success. Thank You!*

**Practitioner Qualitative Interview Guide: Follow-up**

*Thank you for taking the time to share your opinions and experience with me. The following interview is intended to collect information from practitioners involved in Project Reach. The questions that follow will include items about yourself, your practice and how you interact with patients/clients. If you have any additional questions or comments, please feel free to share them with me. This interview will take approximately 30-45 minutes. We are also asking for your permission to audio tape this conversation. You can still participate in this interview if you decline to allow this conversation to be audio taped. Audio taping allows us to make a transcript, which is a more accurate record of our conversation. We then analyze the transcript to determine the results of the study. Any information that might identify you will be deleted from both the written and/or audio record. After the audio tape has been transcribed, we will destroy it. Can I have your permission to audiotape this conversation? <If participant declines, do not begin audio taping. If participant agrees, begin audio taping>.*

Update for second interview:

This line of questions will emphasize what has changed since the last interview. They will explore and test the notion of ramping up, and perhaps ramping down. For example, are the below items becoming easier to do or more difficult? How have you refined your approach? What has become your “standard of practice” now, and what approaches do you think have sustainability after the project is complete? Are there approaches (or materials, referrals, etc) they you used in the beginning but are using less now? Why?

This interview will also try and document the quality of conversations/approaches prior to the training, compared to what they are now.

Like the first interview, this interview will also probe for examples, good and bad, of conversations, and may also explore anything of interest that was reported on in the prior interview.

SECTION 1

1. How often do you discuss lifestyle issues with your patients (such as diet, exercise, tobacco, alcohol, sleep, etc.)?

Prompt: What is most common? How do you go about starting these conversations?

How do your patients react when you talk with them about lifestyle issues?

1. During training, we taught you some strategies – identifying readiness, motivators and barriers -- have you used them for discussing lifestyle issues? How did it work?

Prompt: Which ones were you most comfortable with? Least comfortable with?

How did the patient/client react? Is there anything about your interactions with patients when using these strategies that surprised you?

1. How do you currently document tobacco use?

Prompt: Has this changed recently or since the training? Do you think patients expect to hear about tobacco from their practitioner?

1. After training have you tried to fit conversations about tobacco into your practice?
   1. If YES, continue.

Prompt: If you were already addressing tobacco use before the training, how has anything changed post-training?

At what point are you now introducing the tobacco discussion with your patients?

If applicable, compared to before?

How about e-cigs?

Does this differ by type of patient – your relationship with them, their illness, how much they say they smoke?

Opening lines?

What would it take to keep you involved in talking about tobacco to clients?

Do you approach tobacco differently than other lifestyle issues (diet, nutrition, exercise)?

Why or why not different?

Has this changed due to their participation in project?

Have you had conversations within social network and how did those go?

**If NO, skip to SECTION 4**

SECTION 2

1. Any positive and/or negative interactions? What advice/materials did you offer (referrals, services, medication handout, etc.)? How have you followed up/recovered?

Prompt: How did you go about starting the conversation? How easy or comfortable? How difficult or uncomfortable?

Have you engaged/encouraged patients to do a quit plan, why or why not?

Did you refer anyone to ASHLine…via quitfax or just used the handout? Why one or the other?

Would it help if we provided more copies of Quitfax?

Did you use medications handout, why or why not?

E-cig hand out and in what context?

In addition to what the training offered, are you doing anything to help clients with tobacco cessation?

What therapies are you using to alleviate symptoms of tobacco if any?

FOR MOBILE PRACTITIONERS ONLY: What materials do you travel with? How do you set up? Mobile versus non-mobile practice if they have both – what’s different in going to client home versus if they come to practice (relationship, what they talk about, etc)?

1. During training, we taught you some strategies – identifying readiness, motivators and barriers -- have you used them for tobacco? How did it work?

Prompt: Which ones were you most comfortable with? Least comfortable with?

How did the patient/client react?

Is there anything about your interactions with patients when using these strategies that surprised you?

1. How have the posters/pamphlets worked in making you more comfortable with brining up tobacco? Have patients brought it up themselves?

Prompt: How did you fit these changes into your practice to make them work?

How are you integrating the project handouts with what you have already been doing for tobacco cessation? E-cigs (if applicable)?

How have your patients/clients reacted to the new materials?

How have the posters/pamphlets helped your conversations with new patients?

How have the posters/pamphlets’ helped your conversations with established patients?

Do you plan to keep these materials out after the project ends?

1. Have more smokers entered your practice since you took part in the training? Did you seek them out or did they find you through referral from others?

Prompt: How have you advertised yourself as a resource for tobacco cessation since taking part in the training? Do you see yourself continuing to do so?

1. Were you concerned that bringing up tobacco may be uncomfortable for patients and could harm the practice? Is this still a concern? Why (or why not)?
2. Have you had a conversation with someone who was concerned about a friend or family member who smokes? If YES, How did that conversation get started?

Prompts if needed: How easy or difficult?

If you talked about health effects of tobacco, what specifically did you address?

How did the patient/client react?

Did you refer clients to Helpers?

How comfortable are you in referring clients to Helpers?

IF NO, how can SHS exposure questioning be prioritized higher in your practice? Would you add SHS exposure question to intake?

SECTION 3

Now I’d like to ask you a few questions about the Reach Training…

Look at Qual Data:

1. What parts of the training were most useful? Least?

Prompt: How has the training affected the way you talk to patients? (specifically, about behavior change)? How have you changed they way you offer advice about tobacco since the training?

What could the training have better prepared you for?

In regards to SHS conversations, what could we have done or provided in the training to better help you integrate it into your practice?

How can the Reach team better support you to integrate tobacco helping conversations into your practice?

1. What materials provided at the training were most useful? Least? Is there anything else you would like to have?

Prompt: What handouts did you distribute the most? The least?

Was it better to have a variety of handouts, allowing you to pick and choose the ones you liked? Does having these materials make talking about tobacco seem more like part of your role as a practitioner?

Do you plan to keep these materials out after the project ends?

1. What advice would you give other practitioners who have gone through the training? Have you shared the training with other practitioners?

Prompt: How does your support of tobacco cessation impact the way others look at your profession?

Do you feel your profession has a public health as well as a medical/healthcare role?

If yes --what do you see as the public health contribution of your profession?

How do you think smoking cessation could be an important part of your profession’s public health agenda?

Definition of PH: It is the practice of preventing disease and promoting good health within groups of people.

Now I would like to ask you some questions about yourself….

1. Do you currently smoke or use tobacco? Can you please describe your smoking/tobacco use to me? How has participating in this study influenced your tobacco use? Quit attempts?

SECTION 4

IF NOT having conversations or are using passive approach:

1. Why aren’t you having them? Concerns about offending patients?

Prompt: Do they approach tobacco differently that other lifestyle issues (diet, nutrition, exercise)? Why or why not different?

1. Is this something you think you will do in the future? Are you confident/comfortable that you could do this if/when you decided to start?
2. What do you think will get you to have these conversations?

Prompt: Once started what would it take to keep you involved in having these conversations?

1. If materials are around, to what extent are you waiting for patients to approach you?

Now I’d like to ask you a few questions about the Reach Training…

1. What parts of the training were most useful? Least?

Prompt: How has the training affected they way you talk to patients? (specifically, about behavior change)?

How have you changed they way you offer advice about tobacco since the training? What could the training have better prepared you for?

In regards to SHS conversations, what could we have done or provided in the training to better help you integrate it into your practice?

How can the Reach team better support you to integrate tobacco helping conversations into your practice?

1. What materials provided at the training were most useful? Least? Is there anything else you would like to have?

Prompt: What handouts did you distribute the most? The least?

Was it better to have a variety of handouts, allowing you to pick and choose the ones you liked? Does having these materials make talking about tobacco seem more like part of your role as a practitioner?

Do you plan to keep these materials out after the project ends?

1. What advice would you give other practitioners who have gone through the training? Have you shared the training with other practitioners?

Prompt: How does your support of tobacco cessation impact the way others look at your profession?

Do you feel your profession has a public health as well as a medical role?

If yes --what do you see as the public health contribution of your profession?

How do you think smoking cessation could be an important part of your professions public health agenda?

Now I would like to ask you some questions about yourself….

1. Do you currently smoke or use tobacco? Can you please describe your smoking/tobacco use to me? How has participating in this study influenced your tobacco use? Quit attempts?

*This concludes my questions. Before we end, are there any final thoughts or comments you about your experiences with patients in your office that you would like to add?*

*Thank you very much for your time and input. You will receive your compensation from this interview in about 6-8 weeks. This is a ground-breaking study, and your participation is critical to the study’s success. Thank You!*
